# An adenovirus serotype 2-vectored ebolavirus vaccine generates robust antibody and cell-mediated immune responses in mice and rhesus macaques

**DOI:** 10.1038/s41426-018-0102-5

**Published:** 2018-06-06

**Authors:** Yupeng Feng, Chufang Li, Peiyu Hu, Qian Wang, Xuehua Zheng, Yongkun Zhao, Yi Shi, Songtao Yang, Changhua Yi, Ying Feng, Chunxiu Wu, Linbing Qu, Wei Xu, Yao Li, Caijun Sun, Fu Geroge Gao, Xianzhu Xia, Liqiang Feng, Ling Chen

**Affiliations:** 1 State Key Laboratory of Respiratory Disease, Guangzhou Institutes of Biomedicine and Health, Chinese Academy of Sciences, Guangzhou, 510530 China; 2 University of Chinese Academy of Sciences, Beijing, 100049 China; 3 The Guangzhou 8th People’s Hospital, The First Affiliated Hospital of Guangzhou Medical University, Guangzhou, 510060 China; 4 Key Laboratory of Jilin Province for Zoonosis Prevention and Control, Institute of Military Veterinary, Academy of Military Medical Sciences, Changchun, 130122 China; 5 CAS Key Laboratory of Pathogenic Microbiology and Immunology, Institute of Microbiology, Chinese Academy of Sciences, Beijing, 100101 China

## Abstract

Ebolavirus vaccines based on several adenoviral vectors have been investigated in preclinical studies and clinical trials. The use of adenovirus serotype 2 as a vector for ebolavirus vaccine has not been reported. Herein, we generated rAd2-ZGP, a recombinant replication-incompetent adenovirus serotype 2 expressing codon-optimized Zaire ebolavirus glycoprotein, and evaluated its immunogenicity in mice and rhesus macaques. rAd2-ZGP induced significant antibody and cell-mediated immune responses at 2 weeks after a single immunization. The glycoprotein (GP)-specific immune responses could be further enhanced with a booster immunization. Compared to protein antigens, Zaire ebolavirus GP and Zaire ebolavirus-like particles, rAd2-ZGP could induce stronger cross-reactive antibody and cell-mediated immune responses to heterologous Sudan ebolavirus in mice and rhesus macaques. In rAd2-ZGP-immunized macaques, GP-specific CD8^+^ T cells could secret IFN-γ and IL-2, indicating a Th1-biased response. In adenovirus serotype 5 seropositive macaques, rAd2-ZGP could induce robust antibody and cell-mediated immune responses, suggesting that the efficacy of rAd2-ZGP is not affected by pre-existing immunity to adenovirus serotype 5. These results demonstrated that rAd2-ZGP can be considered an alternative ebolavirus vaccine for use in adenovirus serotype 5 seropositive subjects or as a sequential booster vaccine after the subjects have been immunized with a recombinant adenovirus serotype 5-based vaccine.

## Introduction

Ebolavirus (EBOV) belongs to a genus of the *Filoviridae* family and consists of five species, including Zaire, Sudan, Bundibugyo, Reston, and Taï Forest EBOVs^1^. Among these species, Zaire EBOV (ZEBOV) and Sudan EBOV (SEBOV) have the highest pathogenicity in humans^1^. EBOV infection usually leads to severe hemorrhagic fever diseases with high fatality^2^. The 2013–2016 epidemic caused by ZEBOV in West Africa has become the largest outbreak recorded, with more than 28,000 people affected and a death toll of at least 12,000^3^. The economic and health burdens posed by EBOV highlighted the need to develop safe and effective prophylactic vaccines.

Recombinant adenoviruses (rAd) have been extensively explored as vaccine vectors for many pathogens, including HIV, mycobacterium tuberculosis, malaria, influenza virus, and EBOV^4–7^. rAd vectors expressing EBOV glycoprotein (GP), the major surface protein mediating the attachment and entry of EBOV^8^, effectively protected mice and non-human primates (NHPs) against lethal EBOV infection^6, 9^. At least three rAd-vectored vaccines, including recombinant human adenovirus serotype 5 (rAd5), human adenovirus serotype 26 (rAd26), human adenovirus serotype 35 (rAd35), and chimpanzee adenovirus type 3 (ChAd3), have been investigated^10–13^. Recently, the rAd5-vectored vaccine expressing GP of the ZEBOV Makona strain (GenBank accession number KJ660346) underwent a phase II clinical trial in Sierra Leone and showed good safety and immunogenicity^14^. The rAd5-GP-induced antibody response to EBOV peaked at day 28 after immunization but declined by 85% during the following 6 months^14, 15^, suggesting that a booster immunization later might be necessary. Of particular note, rAd5-GP vaccine-induced GP-specific antibody and T cell responses were weakened by the presence of pre-existing anti-Ad5 antibody in a phase I trial conducted in China and a phase II trial conducted in Sierra Leone, which suggested that pre-existing anti-Ad5 immunity might dampen the immunogenicity of rAd5-vectored EBOV vaccines^14–16^. Therefore, there is a need to evaluate more EBOV vaccines based on other rAd serotypes for immunization in Ad5-seropositive people and for immunization in people who previously received rAd5-vectored vaccines.

Both neutralizing antibody and cell-mediated immune (CMI) responses play important roles in the protection against EBOV infection by rAd-vectored vaccines in rodent and NHP models^17–19^. A strong association has been observed between the titers of GP-specific neutralizing antibodies and the protective efficacy of rAd5-GP in rodent and NHP challenge models^18–20^. It was also demonstrated that rAd5-GP-induced CD8^+^ T cell responses are critical for preventing the establishment of infection through clearing of EBOV-infected host cells in NHPs^13^. Although rAd5 appeared to be a safe and effective vector for an EBOV vaccine, there is a high prevalence of pre-existing anti-Ad5 neutralizing antibodies in human populations, especially in Asia and Africa^16, 21, 22^. We therefore investigated whether recombinant human adenovirus serotype 2 (rAd2) could be used as another adenovirus vector for EBOV vaccines. Ad2 and Ad5 both belong to human adenovirus subgroup C but are distinct in sero-reactivity^23^. rAd2 has been shown to be a safe and efficient vector in humans in many gene therapy clinical trials^24^. Given that pre-existing anti-Ad5 neutralizing antibodies have minimal cross-neutralizing activity to Ad2 and people who are Ad5 seropositive might not be Ad2 seropositive^25^, we proposed that Ad2 could be exploited as another vector for an EBOV vaccine.

In this study, we generated an rAd2 vector carrying a codon-optimized GP gene encoding the contemporary ZEBOV Makona strain (rAd2-ZGP). We investigated the immunogenicity of rAd2-ZGP by assessing GP-specific antibody and CMI responses in mice and NHPs. We also evaluated the immunogenicity of purified GP of ZEBOV (ZGP) and virus-like particles of the ZEBOV Makona strain (ZVLPs).

## Results

### Characterization of rAd2-ZGP, ZGP, and ZVLPs

We generated rAd2-ZGP, a recombinant replication-incompetent adenovirus serotype 2 carrying the GP of the contemporary ZEBOV Makona strain (GenBank accession number KJ660346) (Supplementary Figure S1A). The antigen components in three EBOV vaccine candidates were confirmed by SDS-PAGE followed by western blot analysis as described in the Methods (Supplementary Figure S1B). We estimated that the accumulated level of GP expressed by 5 × 10^8^ vp rAd2-ZGP on cultured Vero cells was ~1 μg (between 0.64 and 1.28 μg) by 72 h post infection (Supplementary Figure S1C). The GP of ZEBOV 2014 (ZGP) with a truncated mucin-like domain and a transmembrane domain (~75 kDa) was produced in Sf9 cells using the baculovirus expression system. The ZVLPs were generated by expressing full-length GP and VP40 of the ZEBOV Makona strain. Under electron microscopy, these filamentous ZVLPs were ~70 nm in diameter and 500–1000 nm in length, resembling the filovirus particles in size and morphology (Supplementary Figure S1D).

### rAd2-ZGP elicited specific antibody responses to homologous ZEBOV and heterologous SEBOV in mice

**Fig. 1:**
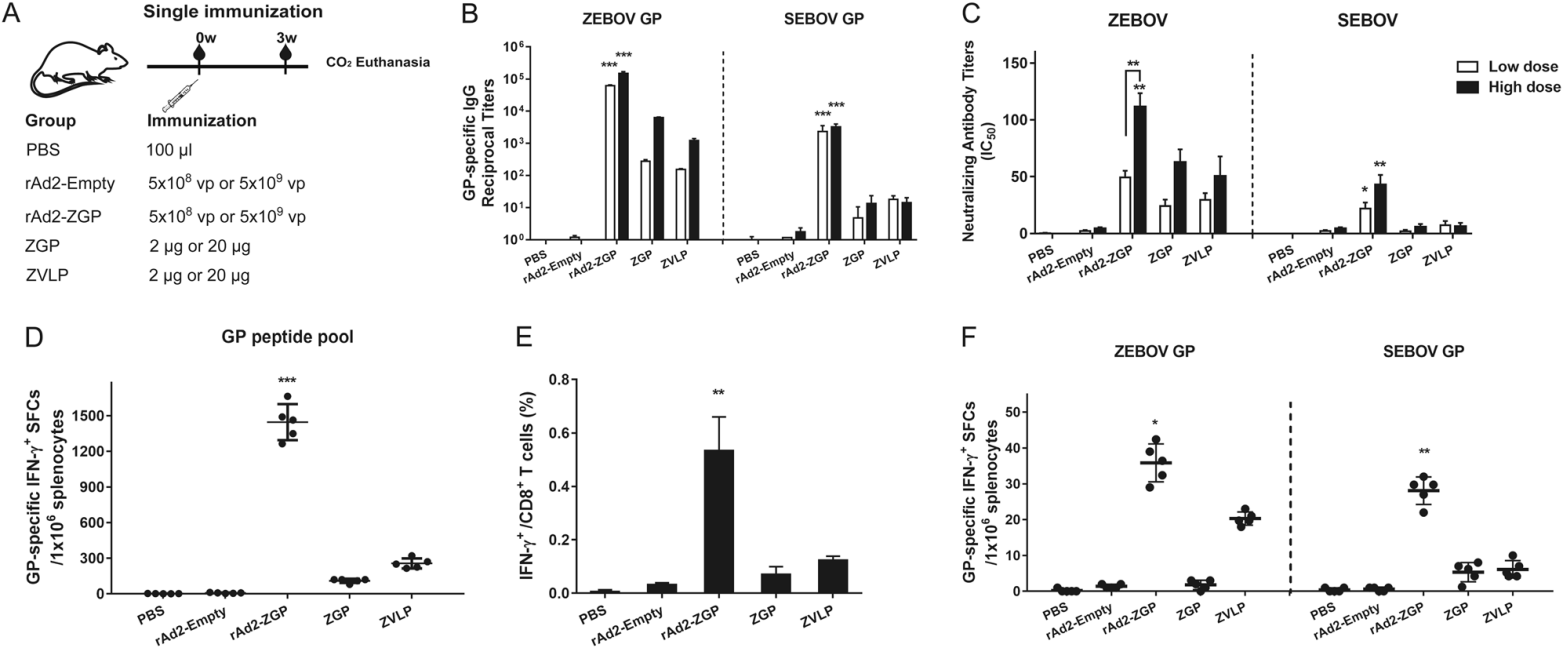
Antibody and cell-mediated immune responses to homologous ZEBOV and heterologous SEBOV after a single immunization in mice. **a** Seven-week-old Balb/c female mice were intramuscularly immunized with 5 × 10^8^ vp rAd2-ZGP, 5 × 10^9^ vp rAd2-ZGP, 2 µg ZGP, 20 µg ZGP, 2 µg ZVLPs, or 20 µg ZVLPs. Mice injected with PBS, 5 × 10^8^ vp or 5 × 10^9^ vp rAd2-Empty were used as control groups. **b** Three weeks after immunization, serum samples were collected and subjected to ELISA analysis of IgG antibodies that bind to ZEBOV GP and SEBOV GP. The titers were calculated as reciprocal endpoints. A cutoff value for a positive result was calculated as the mean optical density (at a 1:100 dilution) for the control serum sample plus 3 SDs. **c** Three weeks after immunization, the serum samples were measured for the neutralizing activities to ZEBOV GP pseudo-typed lentivirus or SEBOV GP pseudo-typed lentivirus. Pseudo-typed lentiviruses at 100 TCID_50_ were incubated with 8 serial twofold dilutions of serum samples from each group and infected into Huh-7 cells. The neutralizing activity was measured as the decrease of luciferase expression relative to negative control sera. The IC50 was calculated by the dose–response inhibition function in GraphPad Prism 7.00. Data are presented as the mean ± SD (*n* = 5). **d** Mice immunized with higher dosage groups were sacrificed 3 weeks after immunization. Splenocytes were isolated and stimulated with a peptide pool derived from ZEBOV GP. IFN-γ^+^ SFCs were assessed with an ELISpot assay and imaged with an ELISpot reader. Data are shown as the number of SFCs in one million splenocytes. **e** CD8^+^ T cells secreting IFN-γ were determined with an ICS assay after stimulation with a peptide pool derived from ZEBOV GP. Data are shown as the mean ± SD (*n* = 5). **f** Splenocytes were stimulated with either ZEBOV GP or SEBOV GP. IFN-γ^+^ SFCs were assessed with an ELISpot assay. Data are shown as the number of SFCs in one million splenocytes. Comparisons between groups were performed by one-way ANOVA, comparisons of the neutralizing antibody titers between low- and high-dose immunizations in the rAd2-ZGP group were performed by Student’s *t* test, and a *P*-value < 0.05 was considered statistically significant. *, *P* < 0.05; **, *P* < 0.01; ***, *P* < 0.001

To evaluate whether rAd2-ZGP can elicit GP-specific antibody responses, female Balb/c mice were intramuscularly immunized with either a lower dosage of 5 × 10^8^ vp rAd2-ZGP or a higher dosage of 5 × 10^9^ vp rAd2-ZGP. Mice injected with either 2 µg ZGP, 20 µg ZGP, 2 µg ZVLPs, or 20 µg ZVLPs were evaluated in parallel. Mice injected with either 5 × 10^8^ vp rAd2-Empty, 5 × 10^9^ vp rAd2-Empty, or PBS were used as mock controls (Fig. 1a). The antibody response was evaluated at 3 weeks after the single immunization. Although 5 × 10^8^ vp rAd2-ZGP is estimated to produce ~1 µg GP in cultured Vero cells, it generated a much higher level of GP-binding IgG antibodies to autologous ZEBOV GP than both the 2 and 20 µg ZGP and ZVLPs. We also assessed if the GP-specific antibody response has any cross-reactivity to heterologous SEBOV GP. Although ZEBOV GP shares 67% amino-acid identity with SEBOV GP (Supplementary Figure S1E), rAd2-ZGP could elicit significant cross-reactive antibodies to heterologous SEBOV GP (Fig. 1b). To further assess the quality of GP-specific antibodies, we measured the neutralizing antibodies using a neutralization assay based on reporter lentiviruses pseudo-typed by ZEBOV GP or SEBOV GP. A single immunization with rAd2-ZGP elicited a significantly higher level of neutralizing antibodies to ZEBOV than did the ZGP and ZVLP vaccines (Fig. 1c, Supplementary Figure S2). A single immunization with rAd2-ZGP, but not ZGP or ZVLPs, induced apparent cross-reactive neutralizing antibodies to heterologous SEBOV (Fig. 1c, Supplementary Figure S2). Both GP-specific binding antibodies and neutralizing antibodies increased with a higher dosage of immunization (Fig. 1b, c, Supplementary Figure S2).

**Fig. 2:**
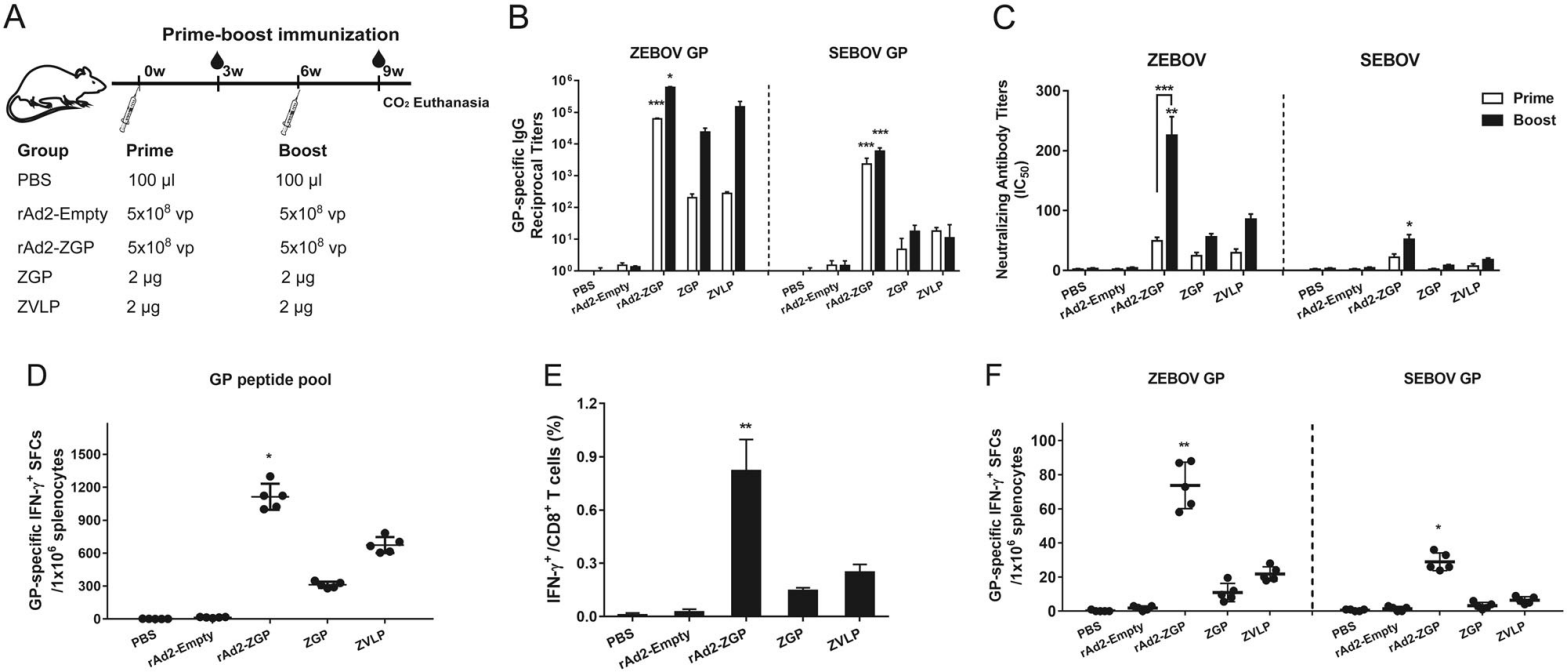
Antibody and cell-mediated immune responses to homologous ZEBOV and heterologous SEBOV after prime-boost immunization in mice. **a** Seven-week-old Balb/c female mice were intramuscularly injected with 5 × 10^8^ vp rAd2-ZGP, 2 µg ZGP, or 2 µg ZVLPs. Six weeks later, mice received a booster immunization of the same vaccine. Mice injected with PBS or 5 × 10^8^ vp rAd2-Empty were used as control groups. **b** Three weeks after each immunization, serum samples were collected and subjected to ELISA analysis of IgG antibodies that bind to ZEBOV GP and SEBOV GP. The titers were calculated as reciprocal endpoints. A cutoff value for a positive result was calculated as the mean optical density (at a 1:100 dilution) for the control serum sample plus 3 SDs. **c** Three weeks after each immunization, serum samples were measured for neutralizing activities to the ZEBOV GP pseudo-typed lentivirus or SEBOV GP pseudo-typed lentivirus. Pseudo-typed lentiviruses at 100 TCID_50_ were incubated with 8 serial twofold dilutions of serum samples from each group and infected into Huh-7 cells. The neutralizing activity was measured as the decrease of luciferase expression relative to negative sera. The IC50 was calculated by the dose–response inhibition function in GraphPad Prism 7.00. Data are presented as the mean ± SD (*n* = 5). **d** Mice were sacrificed 3 weeks after the booster immunization. Splenocytes were isolated and stimulated with a peptide pool derived from ZEBOV GP. IFN-γ^+^ SFCs were assessed with an ELISpot assay and imaged with an ELISpot reader. Data are shown as the number of SFCs in one million splenocytes. **e** CD8^+^ T cells secreting IFN-γ were determined using an ICS assay after stimulation with a peptide pool derived from ZEBOV GP. Data are shown as the mean ± SD (*n* = 5). **f** Splenocytes were stimulated with either ZEBOV GP or SEBOV GP. IFN-γ^+^ SFCs were assessed with an ELISpot assay. Data are shown as the number of SFCs in one million splenocytes. Comparisons between groups were performed by one-way ANOVA; comparisons of the neutralizing antibody titers between prime and booster immunizations in the rAd2-ZGP group were performed by Student’s *t* test. A *P*-value < 0.05 was considered statistically significant. *, *P* < 0.05; **, *P* < 0.01; ***, *P* < 0.001

We also tested a prime-boost immunization regime using either 5 × 10^8^ vp rAd2-ZGP, 2 µg ZGP, or 2 µg ZVLPs with a booster immunization of the same vaccine 6 weeks after the first immunization. Mice injected with PBS and 5 × 10^8^ vp rAd2-Empty were used as mock controls (Fig. 2a). Although all vaccines generated binding antibodies to ZEBOV GP after the two immunizations, rAd2-ZGP appeared to elicit the strongest IgG antibodies that bind to homologous ZEBOV GP (Fig. 2b). rAd2-ZGP stimulated the strongest neutralizing antibodies to homologous ZEBOV, especially after the booster immunization (Fig. 2c, Supplementary Figure S2C). A booster immunization of rAd2-ZGP induced cross-reactive IgG binding antibodies and neutralizing antibodies to heterologous SEBOV (Fig. 2b, c, Supplementary Figure S2F).

### rAd2-ZGP elicited cell-mediated immune responses to homologous ZEBOV and heterologous SEBOV in mice

The rAd5-vectored EBOV vaccine has been shown to generate CMI responses, which also contribute to the protection against EBOV infection^13, 19^. We therefore examined whether rAd2-ZGP induces CMI responses. Mice immunized with higher dosages (5 × 10^9^ vp rAd2-ZGP, 20 µg ZGP, or 20 µg ZVLPs) were sacrificed at 3 weeks after a single immunization. Mice immunized with lower dosages (5 × 10^8^ vp rAd2-ZGP, 2 µg ZGP, or 2 µg ZVLPs) were sacrificed at 3 weeks after the second immunization. Splenocytes were stimulated with a ZEBOV GP peptide pool. rAd2-ZGP induced significant ZEBOV GP-specific IFN-γ^+^ spot forming cells (SFCs). In contrast, ZVLPs only induced weak ZEBOV GP-specific IFN-γ^+^ SFCs, and ZGP induced little ZEBOV GP-specific IFN-γ^+^ SFCs (Figs. 1d and 2d). We also measured IFN-γ^+^ CD8^+^ T cells in mice that received either one immunization of the higher dosage or two immunizations of the lower dosages. rAd2-ZGP induced the most robust GP-specific IFN-γ^+^ CD8^+^ T cell responses compared with ZGP and ZVLPs (Figs. 1e and 2e, and Supplementary Figure S3).

To assess whether the CMI response is cross-reactive to heterologous SEBOV, mouse splenocytes were stimulated with purified ZEBOV GP or SEBOV GP. Although the use of GP protein is not as efficient as that of GP peptides for measuring GP-specific IFN-γ^+^ SFCs, rAd2-ZGP appeared to induce IFN-γ^+^ SFCs responsive to ZEBOV GP, and rAd2-ZGP also elicited, to a less extent, IFN-γ^+^ SFCs responsive to SEBOV GP (Figs. 1f and 2f). In contrast, ZGP and ZVLPs induced no significant IFN-γ^+^ SFCs responsive to SEBOV GP. These results suggested that rAd2-ZGP could elicit strong CMI responses, including IFN-γ^+^CD8^+^ T cell responses to autologous ZEBOV. rAd2-ZGP can also elicit cross-reactive CMI responses to heterologous SEBOV in immunized mice.

### rAd2-ZGP induced robust antibody responses to homologous ZEBOV and heterologous SEBOV in rhesus macaques

**Fig. 3:**
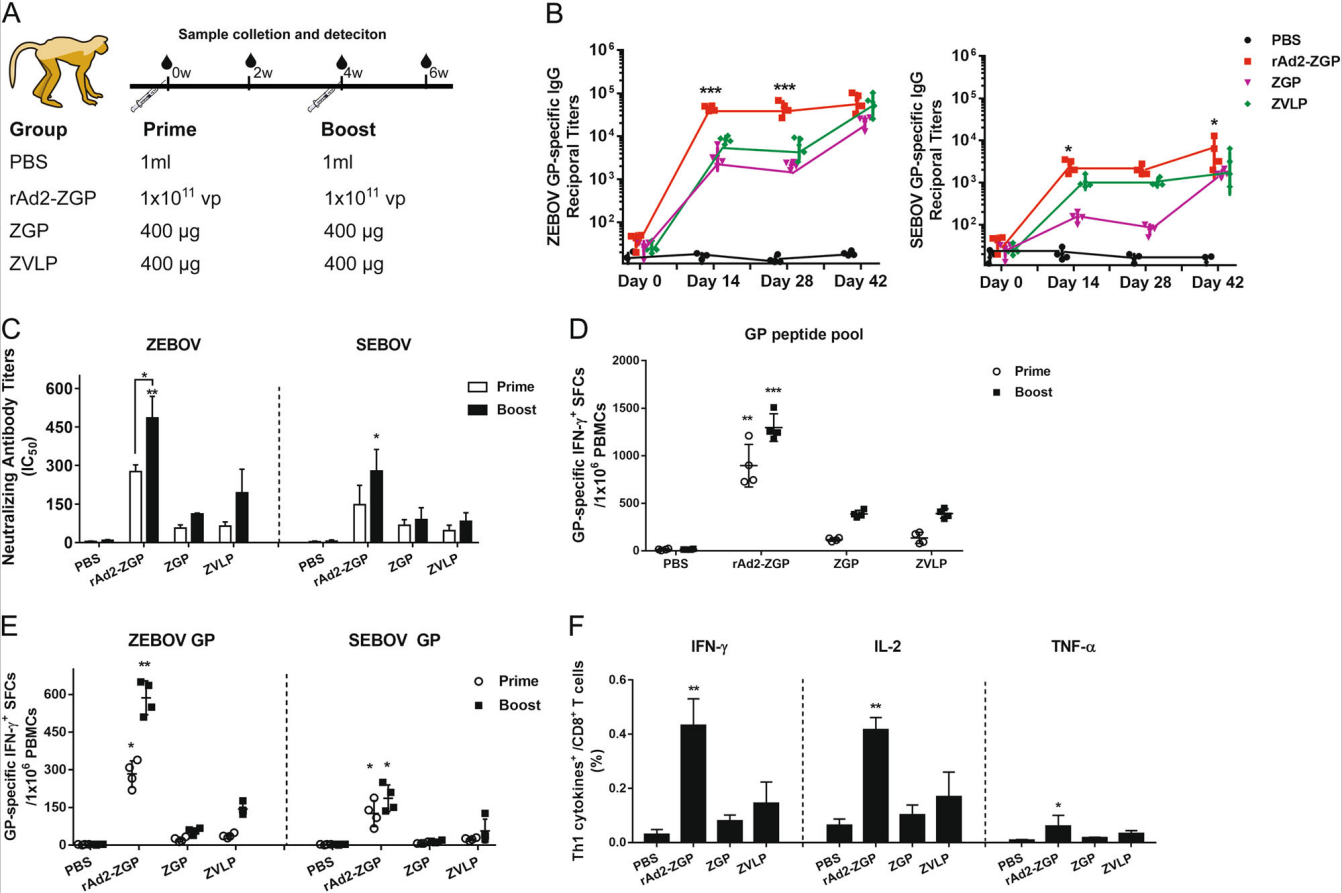
Antibody and cell-mediated immune responses to homologous ZEBOV and heterologous SEBOV after immunization in rhesus macaques. **a** Chinese rhesus macaques were divided into four groups and immunized intramuscularly with either 1 × 10^11^ vp rAd2-ZGP, 400 µg ZGP, 400 µg ZVLPs, or PBS. After 4 weeks, macaques received a booster immunization of the same vaccine. **b** At 0, 2, 4, and 6 weeks after the first immunization, serum samples were collected and subjected to ELISA analysis of IgG antibodies to ZEBOV GP and SEBOV GP. The titers were calculated as reciprocal endpoints. Control serum samples were run each time the assay was performed. A cutoff value for a positive result was calculated as the mean optical density (at a 1:100 dilution) for the control serum sample plus 3 SDs. **c** Serum samples were measured for the neutralizing activities to ZEBOV GP pseudo-typed lentivirus or SEBOV GP pseudo-typed lentivirus. Pseudo-typed lentiviruses at 100 TCID_50_ were incubated with 8 serial twofold dilutions of serum samples from each group and infected into Huh-7 cells. The neutralizing activity was measured as the decrease of luciferase expression relative to negative sera. The IC50 was calculated by the dose–response inhibition function in GraphPad Prism 7.00. Data are presented as the mean ± SD (*n* = 4). **d** PBMCs were collected 2 weeks after the first and second immunizations and were stimulated with a peptide pool derived from ZEBOV GP. IFN-γ^+^ SFCs were assessed with an ELISpot assay. Data are shown as the number of SFCs in one million PBMCs. **e** PBMCs were collected at 2 weeks after each immunization. PBMCs were stimulated with either ZEBOV GP or SEBOV GP. IFN-γ^+^ SFCs were assessed with an ELISpot assay. Data are shown as the number of SFCs in one million PBMCs. **f** Qualitative profiles of ZEBOV GP-specific CD8^+^ T cell responses in PBMCs at 2 weeks after a booster immunization. GP-specific CD8^+^ T cells secreting IFN-γ^+^, IL-2, and TNF-α were determined with ICS analysis. Data are presented as the mean ± SD (*n* = 4). Comparisons between groups were performed by one-way ANOVA; comparisons of the neutralizing antibody titers between prime and booster immunizations in the rAd2-ZGP group were performed by Student’s *t* test. A *P*-value < 0.05 was considered statistically significant. *, *P* < 0.05; **, *P* < 0.01; ***, *P* < 0.001

To evaluate the antibody response elicited by rAd2-ZGP in NHPs, Chinese rhesus macaques were injected intramuscularly with 1 × 10^11^ vp rAd2-ZGP, 400 µg ZGP, 400 µg ZVLPs, or PBS. The same macaque received a booster immunization of the same vaccine 4 weeks after the first immunization (Fig. 3a). rAd2-ZGP rapidly induced a high level of IgG antibodies that bind to ZEBOV GP 2 weeks after one immunization, whereas ZGP and ZVLPs required two immunizations to elicit comparable antibody responses (Fig. 3b, Table 1). rAd2-ZGP generated a high titer of neutralizing antibodies against autologous ZEBOV 2 weeks after one immunization (Fig. 3c, Supplementary Figure S4A, Table 1). Although no significant enhancement in ZEBOV GP-binding antibodies was observed, a booster immunization with rAd2-ZGP further increased the titer of neutralizing antibodies against ZEBOV (Fig. 3c, Supplementary Figure S4B, Table 1). In contrast, ZGP and ZVLPs generated weaker GP-binding antibodies and neutralizing antibodies than did rAd2-ZGP after one immunization. Even after a booster immunization, the neutralizing antibodies elicited by ZGP and ZVLPs were still lower than those by rAd2-ZGP (Fig. 3b, c, Supplementary Figure S4A-B, Table 1). Consistent with the results in mice, rAd2-ZGP also induced a cross-reactive antibody response to SEBOV in rhesus macaques. Binding antibodies to SEBOV GP and neutralizing antibodies to SEBOV were detected in macaques 2 weeks after one immunization with rAd2-ZGP and increased further after a booster immunization, but at a much lower magnitude than to ZEBOV (Fig. 3b, c, Supplementary Figure S4C-D, Table 1). In contrast, ZGP and ZVLPs generated little neutralizing antibodies to SEBOV even after two immunizations (Fig. 3c, Supplementary Figure S4C-D, Table 1). These results demonstrated that rAd2-ZGP can effectively and rapidly generate antibody responses to autologous ZEBOV. rAd2-ZGP can elicit some cross-reactive GP-binding antibodies and neutralizing antibodies to heterologous SEBOV.

**Table 1:** Reciprocal ELISA titers and neutralizing antibody titers 2 weeks after each immunization in rhesus macaques

Group	Day 14				Day 42			
	ELISA IgG titer^a^		nAb titer (IC50)^b^ (range)		ELISA IgG titer		nAb titer (IC50)	
	ZEBOV	SEBOV	ZEBOV	SEBOV	ZEBOV	SEBOV	ZEBOV	SEBOV
PBS	<100	<100	<10	<10	<100	<100	<10	<10
rAd2-ZGP	45,050 ± 5577	2575 ± 790	276 (236–293)	147 (72–253)	68,452 ± 7597	6100 ± 3189	485 (365–560)	278 (187–390)
ZGP	3550 ± 1700	152 ± 36	67 (42–88)	56 (38–66)	22,000 ± 5530	1642 ± 300	110 (89–127)	90 (28–139)
ZVLP	8300 ± 1400	1050 ± 320	64 (48–83)	47 (26–71)	60,800 ± 19,800	3000 ± 2144	193 (84–288)	83 (45–118)
rAd2-ZGP (Ad5 seropositive)	57,650 ± 26,000	3700 ± 717	241 (196–281)	114 (98–124)	80,290 ± 18,000	5675 ± 3220	390 (298–457)	180 (155–215)

^a^The ELISA titers were calculated as reciprocal endpoints. Control serum samples were run each time the assay was performed. A cutoff value for a positive result was calculated as the mean optical density (at a 1:100 dilution) for the control serum sample plus 3 standard deviations

^b^The neutralizing activity was measured as the decrease of luciferase expression relative to negative sera. IC50 was calculated by dose–response inhibition function in GraphPad Prism 7.00

### rAd2-ZGP elicited cell-mediated immune responses to autologous ZEBOV and heterologous SEBOV in rhesus macaques

To evaluate CMI responses elicited by rAd2-ZGP in NHPs, GP-specific enzyme-linked immunospot (ELISpot) assay and intracellular cytokine staining (ICS) assays were performed using freshly isolated peripheral blood mononuclear cells (PBMCs) from these rhesus macaques (Fig. 3a). After the first immunization, macaques immunized with rAd2-ZGP developed the strongest IFN-γ^+^ ELISpot response to ZEBOV GP peptides, which were further enhanced after a booster immunization, whereas macaques immunized with ZGP and ZVLPs only generated a weak IFN-γ^+^ ELISpot response after a booster immunization (Fig. 3d). To assess whether the CMI response was cross-reactive to heterologous SEBOV, macaque PBMCs were stimulated with either autologous ZEBOV GP or heterologous SEBOV GP protein. rAd2-ZGP generated IFN-γ^+^ SFCs responsive to ZEBOV GP, especially after a booster immunization (Fig. 3e). rAd2-ZGP also induced, but to a less extent, IFN-γ^+^ SFCs responsive to SEBOV GP. In contrast, ZGP and ZVLPs induced no significant cross-reactive CMI response to SEBOV GP. Given that antigen-specific CD8^+^ T cells secreting cytokines play important roles in the clearance of EBOV-infected cells^13, 18, 26^, we further analyzed ZEBOV GP-specific CD8^+^ T cells for the production of Th1 cytokines, including IFN-γ, IL-2, and TNF-α, by an ICS assay using a flow cytometer. Macaques immunized with rAd2-ZGP produced ZEBOV GP-specific CD8^+^ T cells producing IFN-γ, IL-2, and TNF-α (Fig. 3f, Supplementary Figure S5). In contrast, macaques immunized with ZGP and ZVLPs generated IFN-γ-, IL-2-, and TNF-α-secreting CD8^+^ T cells at much lower levels (Fig. 3f, Supplementary Figure S5). We also measured IFN-γ^+^ ELISpot responses to ZEBOV GP peptides, ZEBOV GP, and SEBOV GP in macaques immunized with rAd2 vector carrying an unrelated Zika virus antigen, rAd2-zika, and we found no significant GP-specific CMI response in macaques immunized with rAd2-zika (Supplementary Figure S6).

Taken together, these results demonstrate that rAd2-ZGP could elicit significant CMI responses against autologous ZEBOV and generate cross-reactive CMI responses to heterologous SEBOV.

### rAd2-ZGP could induce antibody and cell-mediated immune responses in Ad5-seropositive rhesus macaques

**Fig. 4:**
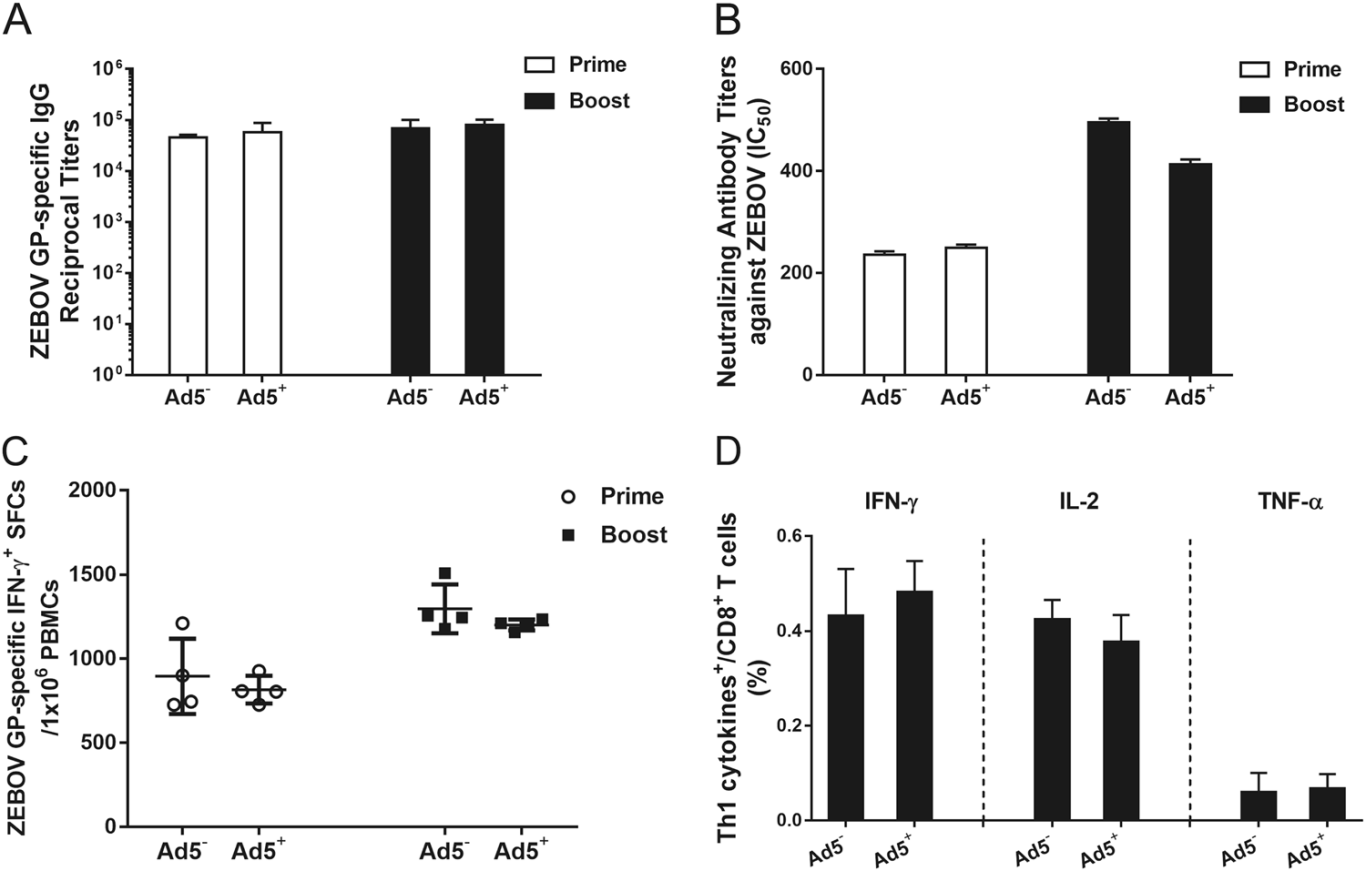
Antibody and cell-mediated immune responses to ZEBOV induced by Ad2-ZGP in Ad5-seropositive rhesus macaques. Chinese rhesus macaques seropositive for Ad5 neutralizing antibodies were immunized intramuscularly with 1 × 10^11^ vp rAd2-ZGP. After 4 weeks, these macaques received a booster immunization of the same vaccine. **a** Serum samples were collected and subjected to ELISA analysis of IgG antibodies that bind to ZEBOV GP. The titers were calculated as reciprocal endpoints. Control serum samples were run every time the assay was performed. A cutoff value for a positive result was calculated as the mean optical density (at a 1:100 dilution) for the control serum sample plus 3 SDs. **b** Serum samples were measured for the neutralizing activities to ZEBOV GP pseudo-typed lentivirus. Pseudo-typed lentiviruses at 100 TCID_50_ were incubated with 8 serial twofold dilutions of serum samples from each group and infected into Huh-7 cells. The neutralizing activity was measured as the decrease of luciferase expression relative to negative sera. The IC50 was calculated by the dose–response inhibition function in GraphPad Prism 7.00. Data are presented as the mean ± SD (*n* = 4). **c** PBMCs were collected 2 weeks after each immunization. PBMCs were stimulated with a peptide pool derived from ZEBOV GP. IFN-γ^+^ SFCs were assessed with an ELISpot assay. Data are shown as the number of SFCs in one million PBMCs. Data are presented as the mean ± SD (*n* = 4). **d** Qualitative profiles of ZEBOV GP-specific CD8^+^ T cell responses in PBMCs 2 weeks after a booster immunization. GP-specific CD8^+^ T cells secreting IFN-γ, IL-2, and TNF-α were determined with ICS analysis. Data are presented as the mean ± SD (*n* = 4)

To investigate whether rAd2-ZGP is effective in inducing GP-specific immune responses in subjects who have pre-existing anti-Ad5 neutralizing antibodies, rhesus macaques that had been previously immunized with rAd5 were immunized with 1 × 10^11^ vp rAd2-ZGP followed by a booster immunization 4 weeks later. These Ad5-seropositive macaques were injected with Ad5 over 1 year ago and had Ad5 neutralizing antibody (mean titer 1:2630) but little cross-neutralization to Ad2 (mean titer 1:173) before immunization with rAd2-ZGP (Supplementary Table S1). rAd2-ZGP-induced GP-specific antibody and CMI responses were compared with Ad5-seronegative macaques immunized with the same immunization regimen. rAd2-ZGP induced similar levels of GP-specific IgG antibodies and neutralizing antibodies to ZEBOV in Ad5-seropositive macaques as in Ad5-seronegative macaques (Fig. 4a, b). Comparable IFN-γ^+^ SFCs were detected in macaques with or without pre-existing anti-Ad5 neutralizing antibodies (Fig. 4c), suggesting that pre-existing anti-Ad5 immunity did not affect the efficacy of rAd2-ZGP in inducing ZEBOV GP-specific CMI responses. rAd2-ZGP provoked similar CD8^+^ T cell responses with a Th1 cytokine profile of IFN-γ^+^, IL-2, and TNF-α. Pre-existing anti-Ad5 neutralizing antibodies did not dampen the immunogenicity of rAd2-ZGP. Therefore, rAd2-ZGP is similarly effective in inducing GP-specific antibody and CMI responses in both Ad5-seronegative and Ad5-seropositive subjects.

## Discussion

Preventing EBOV epidemic outbreaks requires prophylactic vaccines that rapidly induce protective host immune responses, including neutralizing antibody and cytotoxic lymphocyte responses^17–19^. The trimeric surface GP of EBOV, which mediates viral entry and contains the major epitopes for neutralizing antibodies, has been adopted as the principal antigen for EBOV vaccines, including those based on purified GP^27^, VLPs^28^, DNA vector^29^ and recombinant viral vectors such as recombinant adenoviral vectors and the vesicular stomatitis viral vector^9–11, 14, 30, 31^. These vaccine candidates, tested individually in different laboratories, exhibited varying degrees of immunogenicity and protective efficacy in mice, guinea pigs, and NHPs^32^.

In this study, we constructed rAd2-ZGP, a recombinant replication-incompetent rAd2-vectored EBOV vaccine. We investigated in two animal species the antibody and CMI responses induced by rAd2-ZGP and, in parallel, by two protein-based EBOV vaccines, ZGP and ZVLPs. We found the following: (i) rAd2-ZGP generated robust GP-binding IgG antibody and neutralizing antibody responses to autologous ZEBOV (Figs. 1–3); (ii) rAd2-ZGP elicited robust GP-specific CMI responses with CD8^+^ T cells secreting Th1 cytokines in rhesus macaques, whereas ZGP and ZVLPs induced weak CMI responses to EBOV (Fig. 3); (iii) rAd2-ZGP, but not ZGP or ZVLPs, induced some cross-reactive antibody and CMI responses to heterologous SEBOV (Figs. 1–3); and (iv) pre-existing anti-Ad5 immunity showed no attenuation of the immunogenicity of rAd2-ZGP (Fig. 4).

Replication-incompetent adenovirus vectors showed good performance in inducing antibody and T cell responses, which both play important roles in the protection of rAd-based vaccines against EBOV infection^17–19^. Recently, an rAd5-vectored EBOV vaccine was proved to be safe and immunogenic in clinical trials and was approved by the Chinese FDA (NCT02326194)^14, 15^. However, the declining tendency of the immune response at 6 months after rAd5-GP immunization in vaccinees suggested that repeated immunization might be needed to boost and ensure protection in humans^14^. In this study, we demonstrated that an EBOV vaccine based on rAd2, which has also been used extensively in human gene therapy trials and has good safety records in humans, could induce robust antibody and CMI responses against autologous EBOV in mice and rhesus macaques (Figs. 1–3). These antibody and CMI responses also showed significant cross-reactivity to a heterologous SEBOV (Figs. 1–3). These results suggested that rAd2-ZGP, if proved to be safe and protective in further preclinical and clinical studies, could be a potential candidate vaccine to prevent EBOV infection.

Previous studies suggested that rAd-vectored EBOV vaccines based on different serotypes might exhibit different levels of immunogenicity and therefore might differ in protective efficacy^26, 31^. An rAd5-based EBOV vaccine conferred effective protection against EBOV infection in rhesus macaques^13^, whereas rAd26- or rAd35-based vaccines did not exhibit the same level of protection^31^. One possible explanation is that these adenovirus serotypes have different cell tropisms and might use different cell surface receptors for viral entry, which could affect the target cell preference and therefore the antigen expression and presentation^33^. We selected Ad2 because it shares the same cellular receptor with Ad5 and might thus have similar cell tropism as well as immunogenicity to rAd5-based vaccines^34^. Ad2 and Ad5 belong to adenovirus subgroup C, use the coxsackievirus and adenovirus receptors for virus attachment and use the α_v_β_3_ or α_v_β_5_ integrins for virus internalization^35^. rAd2-ZGP induced high levels of GP-binding and neutralizing antibodies in mice and rhesus macaques, which is comparable to the results in other studies using rAd5-GP^6, 13^. Because this level of immune response showed complete protection against EBOV challenge in the context of an rAd5-based vaccine, rAd2-ZGP might have similar protective effects against EBOV infection^6, 9, 13, 18^. The antibody and CMI responses induced by rAd2-ZGP also showed some cross-reactivity to heterologous SEBOV, which has not been reported for rAd5-vectored EBOV vaccines. These features can be valuable for vaccines to be used in an EBOV epidemic region with multiple circulating strains^36^. Importantly, the rapid generation of protective immune responses rendered rAd2-ZGP suitable for pre- and post-exposure immunization in the case of an EBOV outbreak.

There is a report that purified GP provided only partial protection against EBOV infection when given alone or as a booster vaccine with a DNA vaccine^37^, but GP could protect rodents from lethal EBOV challenge when fused with an Fc fragment or formulated as Ebola immune complexes^27, 38^. Our study also showed that the GP protein-based vaccine primarily induced an antibody response that was limited to autologous ZEBOV without significant CMI responses (Figs. 1–3). Further modifications of the protein antigen or its combination with potent adjuvants might be considered to enhance the immunogenicity and protective efficacy of GP protein-based vaccines. ZVLPs, consisting of GP and matrix protein VP40, have been shown to induce EBOV-specific antibody and CMI responses in mice and NHPs^28, 39^. Immunization with a mammalian-derived VLP conferred protection against autologous EBOV in both small animals and NHPs, but much larger dosages, multiple immunizations and the presence of adjuvants were required^39, 40^. In our study, ZVLPs could elicit CMI responses, but the responses were much weaker than those for rAd2-ZGP (Figs. 1–3), possibly due to the low dosage, the limited immunization, and the absence of adjuvants. Therefore, ZVLPs alone cannot elicit efficient protective immune responses after a single immunization and might not be effective in inducing cross-reactive neutralizing antibodies and CMI responses.

Although the exact mechanisms of protection of an effective EBOV vaccine remain to be clarified, it is likely that neutralizing antibody and CMI responses play important roles in controlling viral infection. In this study, we found that a significant proportion of ZEBOV GP-specific CD8^+^ T cells secreting IFN-γ, IL-2, and TNF-α, which have been shown to be associated with protection, were generated in macaques immunized with rAd2-ZGP (Fig. 3). This result highlighted the property of rAd2-ZGP in eliciting Th1-biased T cell responses, which might contribute to the clearance of EBOV-infected host cells.

Pre-existing anti-Ad5 immunity is a concern for the application of rAd5-vectored vaccines because it could attenuate the rAd5-mediated antigen expression and therefore decrease the immunogenicity^41^. In some African countries, ~90% of residents are seropositive for anti-Ad5 neutralizing antibodies^16^. This high seroprevalence might weaken the capacity of rAd5-vectored vaccines in inducing EBOV-specific immunity, as observed in a clinical trial conducted in this population in comparison with other populations with relatively lower anti-Ad5 neutralizing antibody seroprevalence^14, 15^. The rAd2-ZGP described in this study, however, could potentially circumvent the pre-existing anti-Ad5 neutralizing antibodies. rAd2-ZGP induced robust GP-specific neutralizing antibody and CMI responses in macaques with pre-existing anti-Ad5 immunity (Fig. 4). The antibody and CMI responses were comparable to those in Ad5-seronegative macaques. Although Ad2 and Ad5 might co-circulate in populations of developing countries, their neutralizing antibodies do not cross-neutralize each other. People who are seropositive for anti-Ad5 neutralizing antibodies are not always seropositive for anti-Ad2 neutralizing antibodies^25^. Therefore, rAd2-ZGP might be considered an alternative EBOV vaccine candidate for people with pre-existing anti-Ad5 neutralizing antibodies or could be used as a booster vaccine for people who have been previously immunized with an rAd5-based vaccine.

We also noted that a booster immunization with rAd2-ZGP did not significantly increase ZEBOV GP-specific binding antibodies, which was similar to the findings of a previous study by another laboratory^6^. However, a booster immunization with rAd2-ZGP enhanced the titer of neutralizing antibodies against ZEBOV (Fig. 3c), suggesting that a booster immunization can increase the quality of GP-specific antibodies. The booster immunization also significantly increased the cross-reactive binding antibodies to SEBOV GP (Fig. 3b). Moreover, the CMI responses to ZEBOV and SEBOV were increased after a booster immunization (Fig. 3d, e). These results suggested that although the pre-existing anti-Ad2 antibodies might exert some dampening effects on repeated immunization with rAd2-ZGP, the quality of antibody responses and the quantity of CMI responses can still be enhanced.

We generated an rAd2-vectored EBOV vaccine expressing GP and evaluated its immunogenicity in mice and rhesus macaques. We showed that rAd2-ZGP induced robust GP-specific antibody and CMI responses to autologous EBOV, even in the presence of anti-Ad5 immunity. Importantly, immune responses induced by rAd2-ZGP showed cross-reactivity to heterologous SEBOV. The rapid generation of EBOV-specific immune responses renders rAd2-ZGP suitable for pre- and post-exposure immunization in the case of an EBOV outbreak. rAd2-ZGP is worth further investigating in EBOV-challenged NHP models.

## Materials and methods

### Ethics statement

All animal experiments were performed at the Animal Experimental Center of the Guangzhou Institutes of Biomedicine and Health, Chinese Academy of Sciences. The experimental protocols were approved by the Institutional Animal Care and Use Committee (IACUC# 2015006). The Chinese rhesus macaques that participated in this study were free of simian immunodeficiency virus, simian T lymphotropic virus type 1, and simian retrovirus.

### Vaccine preparation

rAd2-ZGP, a replication-incompetent adenovirus type 2 carrying the EBOV glycoprotein-encoding gene, was constructed as previously described^42^. ZGP, the coding sequence for GP from the contemporary ZEBOV Makona strain (GenBank accession number KJ660346), was codon-optimized for mammalian expression and synthesized (Genscript Inc., Nanjing, China). The ZGP gene was placed into the adenovirus shuttle vector pGA1 to obtain pGA1-ZGP. The pGA1-ZGP was linearized and subjected to homologous recombination with the linearized Ad2 backbone with deletion of the E1 and E3 genes in competent *E. coli* BJ5183 cells (Invitrogen, CA, USA). The resultant adenoviral plasmid pAd2-ZGP was linearized and transfected into HEK293 cells to rescue rAd2-ZGP. To generate purified ZGP, the coding sequence corresponding to residues 33–311 and 464–632 of GP from the ZEBOV Makona strain (GenBank accession number KJ660346) was integrated into a baculovirus vector, with a gp67 signal sequence fused to the N-terminus. ZGP protein was expressed in Sf9 cells and purified as previously described^8^. To generate ZVLPs, a recombinant baculovirus expressing full-length GP and VP40 from the ZEBOV Makona strain (GenBank accession number KJ660346) was generated and produced in Sf9 cells^43^. ZVLPs were purified through a discontinuous sucrose gradient (10, 30, and 50%) at 28,000 × *g* for 90 min at 4 °C. For morphology analysis, the ZVLP sample was set on a carbon-coated copper grid, stained with 1% phosphotungstic acid and examined on a Tecnai G2 Spirit (FEI, OR, USA) at an accelerating voltage of 120 kV.

### Western blot analysis of GP

To analyze the rAd2-ZGP-mediated expression of GP, 1 × 10^6^ Vero cells were infected by rAd2-ZGP at 5 × 10^9^ viral particles (vp). At 12, 24, 36, 48, 60, and 72 h post infection, the cells were harvested. One-tenth of the rAd2-ZGP-infected cell lysates and GP standard samples (0.08, 0.16, 0.32, 0.64, 1.28, and 2.56 µg) were subjected to SDS-PAGE followed by western blot analysis. To verify the GP component in the candidate vaccines, the rAd2-ZGP-infected cell lysates at 72 h post infection, purified ZGP and ZVLPs were subjected to SDS-PAGE followed by western blot analysis with anti-GP polyclonal antibodies (Sino Biological, China) and anti-VP40 antibodies (Abcam, MA, USA).

### Evaluation of immunogenicity of vaccine candidates in mice

Seven-week-old female Balb/c mice were injected intramuscularly with two immunization strategies as follows: (1) single immunization with either a lower dosage or a higher dosage of each vaccine candidate, including rAd2-ZGP (5 × 10^8^ vp or 5 × 10^9^ vp per mouse in phosphate-buffered saline, PBS), ZGP (2 or 20 µg per mouse with alum), ZVLPs (2 or 20 µg per mouse in PBS), and rAd2 empty vector (rAd2-Empty, 5 × 10^8^ vp or 5 × 10^9^ vp per mouse in PBS), and (2) prime-boost immunization with a lower dosage of each vaccine candidate, including rAd2-ZGP (5 × 10^8^ vp per mouse in PBS), ZGP (2 µg per mouse with alum), ZVLPs (2 µg per mouse in PBS), and rAd2-Empty (5 × 10^8^ vp per mouse in PBS). At 3 weeks after the first immunization, blood samples were collected. Mice receiving a single immunization with the higher dosages were euthanatized for isolation of splenocytes for further analysis. At 6 weeks after the first immunization, mice receiving a lower dosage of vaccine candidates were boosted similarly as with the first immunization. At 3 weeks after the booster immunization, the mice were euthanatized. Serum samples were collected, and splenocytes were isolated for further analysis.

### Evaluation of immunogenicity of vaccine candidates in rhesus macaques

Twenty 4–6-year-old Chinese rhesus macaques were randomly divided into five groups with both male and female macaques in each group. Each group received two intramuscular vaccinations at 4-week intervals as follows: (1) four macaques received 1 ml PBS as a mock treatment control; (2) four macaques who had not been exposed to adenovirus received two doses of rAd2-ZGP (1 × 10^11^ vp in PBS); (3) four Ad5-seropositive macaques, who were immunized with rAd5 over 1 year ago, received two doses of rAd2-ZGP (1 × 10^11^ vp in PBS); (4) four macaques received two doses of purified ZGP (400 µg in PBS with alum); and (5) four macaques received two doses of ZVLPs (400 µg in PBS). Blood samples were collected, and the serum samples and peripheral blood mononuclear cells were isolated for further analysis as previously described^44^. Macaques receiving rAd2-ZGP were tested for serum neutralizing antibodies to Ad2 and Ad5 before and after immunization of rAd2-ZGP.

### Enzyme-linked immunosorbent assay

EBOV GP-specific binding antibodies were analyzed by enzyme-linked immunosorbent assay (ELISA) as described previously^45^. In brief, 96-well flat-bottom plates were coated with 100 µl purified EBOV GP at 1 µg/ml in PBS at 4 °C overnight. After washing and blocking, serum samples were serially diluted in twofold increments and added in duplicate. After incubation at room temperature for 2 h, the plates were washed, and HRP-labeled secondary antibody was added (Proteintech, IL, USA). After incubation for another 1 h at room temperature, the plates were washed and developed with TMB/E substrate (Merck Millipore, MA, USA). Finally, the reaction was stopped, and the OD_450_ values were read. The ELISA titers were calculated as a reciprocal endpoint. A negative serum control was run each time the assay was performed. A cutoff value for a positive result was calculated as the mean optical density (at a 1:100 dilution) for the negative sera plus 3 standard deviations (SDs).

### Micro-neutralization assay

The micro-neutralization (MN) assays were performed using lentivirus pseudo-typed with EBOV GP as previously described^45^. In brief, EBOV GP pseudo-typed lentiviruses were prepared by co-transfection of the HIV-1 proviral vector pNL4–3.Luc.R^-^E^−^ and expression vectors encoding GP from the ZEBOV Makona strain (GenBank accession number KJ660346) or GP from SEBOV (GenBank accession number KR063670) into 293T cells. Subsequently, the pseudo-typed viruses were harvested, titered, and then mixed with serially diluted serum samples at 100 TCID_50_ per well. After incubation at 37 °C for 30 min, 90–100% confluent Huh-7 cells were infected with the mixture for 4 h. Then, the infection mixture was replaced with complete DMEM medium. After 48 h of incubation, the luciferase activity was detected with a Luciferase Assay System (Promega, WI, USA). The neutralizing activity was measured as the decrease of luciferase expression relative to negative control sera. The half maximal inhibitory concentration (IC50) values were then calculated as the reciprocal of the serum dilution at which 50% neutralization was achieved using GraphPad Prism 7.00^13^.

The titers of neutralizing antibodies against Ad5 and Ad2 were detected with an MN assay as previously described^46^. Briefly, HEK293 cells were seeded into 96-well plates at 3 × 10^4^ cells per well. After 24 h, serial dilutions of heat-inactivated serum samples were incubated with Ad2-SEAP or Ad5-SEAP at 4 × 10^6^ vp per well for 1 h at 37 °C. The mixtures were added to the 96-well plates and incubated for 24 h at 37°C. Subsequently, supernatants were harvested, and the SEAP activity was detected using a Phospha-Light System according to the manufacturer’s instructions (Thermo Fisher Scientific, IL, USA). The relative light units (RLUs) were recorded, and the neutralizing titers were calculated as the reciprocal dilutions that inhibited 50% of the RLU values.

### ELISpot assay

IFN-γ ELISpot assays were performed using freshly isolated splenocytes or PBMCs. In brief, sterile 96-well microtiter plates (Merck Millipore, MA, USA) were coated with IFN-γ coating antibody (R&D Systems, MN, USA) at 4 °C overnight. The plates were then washed and blocked at 37 °C for 2 h. Mouse splenocytes or monkey PBMCs were isolated using a density gradient medium (Lymphoprep^TM^, Vancouver, Canada), seeded at 5 × 10^5^ cells per well and stimulated with a peptide pool of GP from the ZEBOV Makona strain (GenBank accession number KJ660346) (Chinese Peptide Company, China) at 2 µg/ml per peptide or with ZEBOV GP or SEBOV GP (GenBank accession number AKB09538) (Sino Biological, China) at 20 µg/ml. After incubation for 24 h, the plates were incubated with biotinylated detection antibodies and developed with alkaline phosphatase-conjugated streptavidin (BD PharMingen, CA, USA) and NBT/BCIP reagent (Pierce, IL, USA). Finally, the spots were counted with an ELISpot reader (Bioreader 4000, BIOSYS, Germany).

### ICS assay

ICS assays were performed as previously described^5^. In brief, freshly isolated mouse splenocytes or monkey PBMCs were seeded into 96-well plates (2 × 10^6^ cells per well) and incubated with an EBOV GP peptide pool (2 µg/ml). One hour later, brefeldin A (BD Phamingen, CA, USA) was added and incubated for another 10 h. The cells were then harvested, stained with surface antibodies (CD3-Pacific blue, CD8-APC-Cy7, CD4-FITC; BD Biosciences, CA, USA) for 1 h, then permeabilized and stained with intracellular antibodies (IFN-γ-PE, IL-2-APC, TNF-α-PE-Cy7; BD Biosciences, CA, USA). Finally, the cells were detected with an LSR Fortessa SORP instrument (BD Biosciences, CA, USA).

### Statistical analysis

Flow cytometric data were analyzed using FlowJo version 7.6 (Tree Star, Inc., Ashland, USA). Statistical analyses and graphical presentations were conducted with GraphPad Prism version 6.0 (GraphPad Software, Inc., CA, USA). Differences among groups were tested with one-way ANOVA. Differences in the same vaccine groups were determined by Student’s *t* test. Throughout the text, figures, and legends, the following terminology is used to show statistical significance: *, *P* < 0.05; **, *P* < 0.01; and ***, *P* < 0.001.

### Data availability

The authors declare that all relevant data are available from the corresponding author upon request.

## Electronic supplementary material

Supplementary Table S1

Supplementary Figure S1

Supplementary Figure S2

Supplementary Figure S3

Supplementary Figure S4

Supplementary Figure S5

Supplementary Figure S6
