# Supplementary material for: An adenovirus serotype 2-vectored ebolavirus vaccine generates robust antibody and cell-mediated immune responses in mice and rhesus macaques
Source: Emerg Microbes Infect. 2018 Jun 6;7:101. doi: 10.1038/s41426-018-0102-5 (PMC5988821; doi:10.1038/s41426-018-0102-5)
Supplement: Supplementary file 1 — Supplementary Table S1 [file 41426_2018_102_MOESM1_ESM.pdf]

Supplementary Table S1. Neutralizing antibody titers against Adenovirus serotype 2 and serotype 5 in rAd2-ZGP immunized rhesus macaques.

Group	Day 0 nAb titer (IC50) (range)[a]		Day 28 nAb titer (IC50) (range)		Day 42 nAb titer (IC50) (range)	
	Ad5	Ad2	Ad5	Ad2	Ad5	Ad2
rAd2-ZGP immunization in Ad5 seronegative macaques	<18	<18	843 (24-1536)	18986 (11484-31768)	1587 (314-2983)	64319 (38176-119097)
rAd2-ZGP immunization in Ad5 seropositive macaques	2620 (1331-4608)	173 (18-314)	4934 (4021-7159)	25289 (19028-31253)	8434 (4021-11931)	92897 (41174-198067)

[a]The titers of neutralizing antibodies against Ad5 and Ad2 were detected with a MN assay. The SEAP activity was detected and the relative light units (RLUs) were recorded. The neutralizing titers (IC50) were calculated as the reciprocal dilutions that inhibited 50% RLU values.
