# Supplementary material for: An adenovirus serotype 2-vectored ebolavirus vaccine generates robust antibody and cell-mediated immune responses in mice and rhesus macaques
Source: Emerg Microbes Infect. 2018 Jun 6;7:101. doi: 10.1038/s41426-018-0102-5 (PMC5988821; doi:10.1038/s41426-018-0102-5)
Supplement: Supplementary file 2 — Supplementary Figure S1 [file 41426_2018_102_MOESM2_ESM.pdf]

Supplementary Figure S1

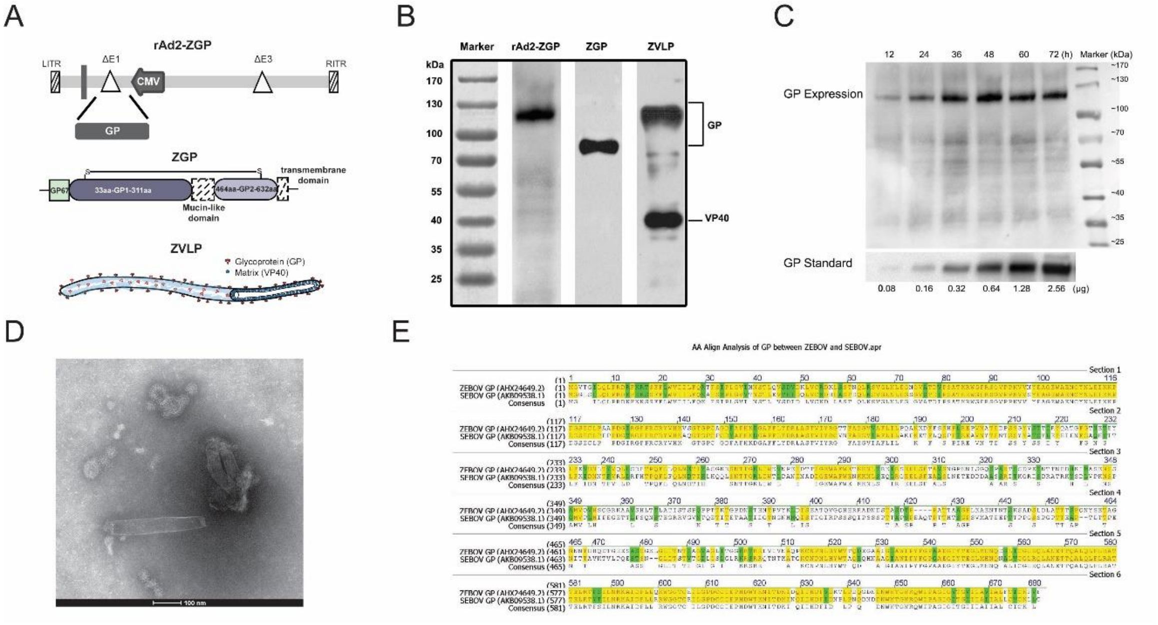

Supplementary Figure S1. Schematic presentation and characterization of EBOV vaccines rAd2-ZGP, ZGP, and ZVLP.

(A) The codon optimized Zaire EBOV *gp* gene was inserted into the deleted E1 region of Ad2 in which the E3 region is deleted. The purified ZGP has a mucin domain deleted GP1 and a transmembrane domain removed. The ZVLP contains full-length EBOV GP and VP40 of Zaire EBOV. (B) rAd2-ZGP infected Vero cells, purified ZGP, and ZVLP were analyzed using SDS-PAGE without β-mercaptoethanol. Western blot analysis was performed using polyclonal anti-GP antibodies (Sino Biological) and a monoclonal antibody specific for EBOV VP40 (Abcam). (C) 1×10^6 Vero cells were infected by rAd2-GP at 5×10^9 viral particles (vp). At 12, 24, 36, 48, 60, and 72 hours post infection, cell lysates were collected at indicated time pointes and the GP contents in one tenth of the collected cell lysates were analyzed by Western blot assays using a polyclonal anti-GP antibody (Sino Biological, China). Quantitative analysis of the GP contents accumulated

in Vero cells were calculated by comparison to the ZGP standard curve. The standard curve of ZGP protein was constructed by plotting the Gray values versus the contents of purified GP protein. (D) TEM picture. ZEBOV GP and EBOV VP40 could form the filamentous particle-like structures, bar=100 nm. (E) Amino acid sequence alignment of GP from ZEBOV Makona strain (Genbank accession number KJ660346) and GP from SEBOV (Genbank accession number KR063670).
