# Supplementary material for: An adenovirus serotype 2-vectored ebolavirus vaccine generates robust antibody and cell-mediated immune responses in mice and rhesus macaques
Source: Emerg Microbes Infect. 2018 Jun 6;7:101. doi: 10.1038/s41426-018-0102-5 (PMC5988821; doi:10.1038/s41426-018-0102-5)
Supplement: Supplementary file 3 — Supplementary Figure S2 [file 41426_2018_102_MOESM3_ESM.pdf]

Supplementary Figure S2

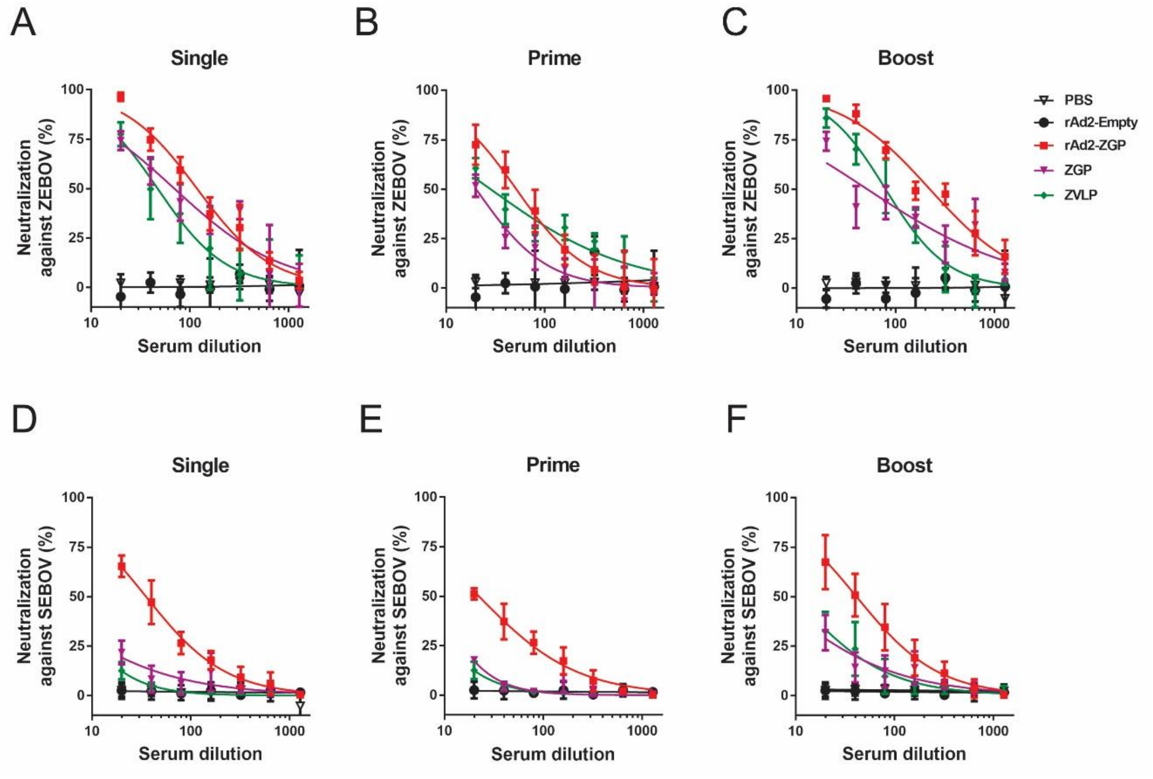

Supplementary Figure S2. Neutralization capability of mouse serum samples against EBOV GP pseudo-typed viruses.

The neutralizing activity of serums from mice was assessed by MN assay based on ZEBOV GP pseudo-typed lentivirus (A-C) or based on SEBOV GP pseudo-typed lentivirus (D-F). 100 TCID50 of pseudo-typed viruses was incubated with dilutions of serum samples and then infected into Huh-7 cells. The neutralization was measured as the decrease in luciferase expression relative to negative serums. The dose-response curves were constructed by four-parameter non-linear regression in GraphPad Prism 7.00. Data were presented as mean ± SD (n=5)
