# Supplementary material for: An adenovirus serotype 2-vectored ebolavirus vaccine generates robust antibody and cell-mediated immune responses in mice and rhesus macaques
Source: Emerg Microbes Infect. 2018 Jun 6;7:101. doi: 10.1038/s41426-018-0102-5 (PMC5988821; doi:10.1038/s41426-018-0102-5)
Supplement: Supplementary file 4 — Supplementary Figure S3 [file 41426_2018_102_MOESM4_ESM.pdf]

Supplementary Figure S3

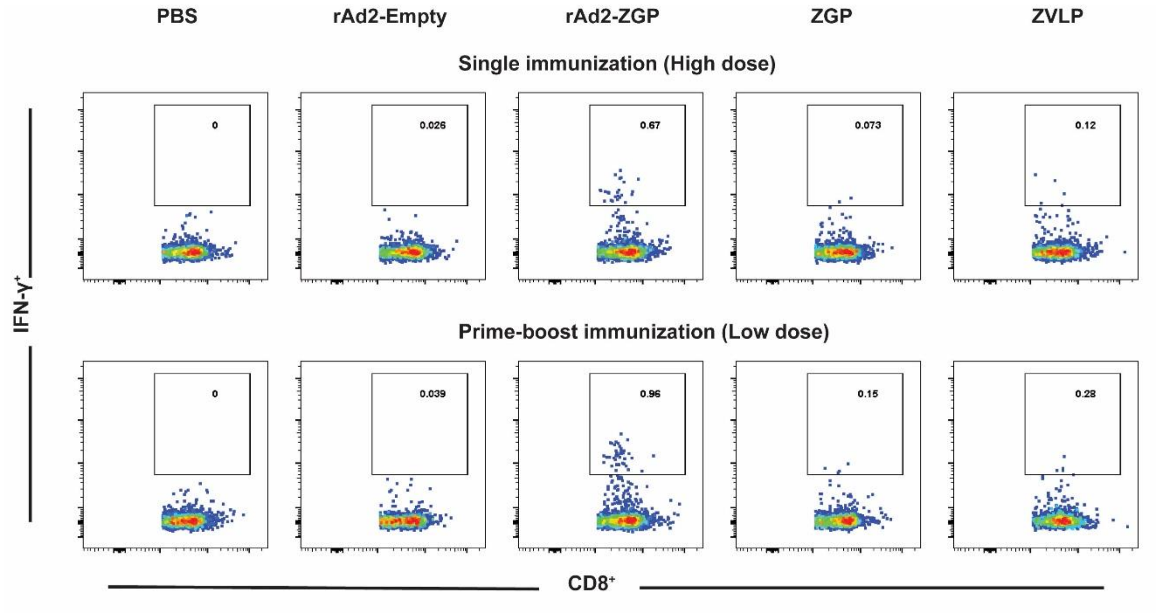

Supplementary Figure S3. IFN-γ$^+$ CD8$^+$ T cell responses in vaccinated mice. Three weeks after the single or prime-boost immunization, mice were sacrificed. Splenocytes were isolated and stimulated with a ZEBOV GP peptide pool and stained for markers (CD3, CD4, CD8 and IFN-γ). The CD8$^+$ T cells were gated and IFN-γ$^+$ CD8$^+$ T cells were shown.
