# Supplementary material for: An adenovirus serotype 2-vectored ebolavirus vaccine generates robust antibody and cell-mediated immune responses in mice and rhesus macaques
Source: Emerg Microbes Infect. 2018 Jun 6;7:101. doi: 10.1038/s41426-018-0102-5 (PMC5988821; doi:10.1038/s41426-018-0102-5)
Supplement: Supplementary file 6 — Supplementary Figure S5 [file 41426_2018_102_MOESM6_ESM.pdf]

Supplementary Figure S5

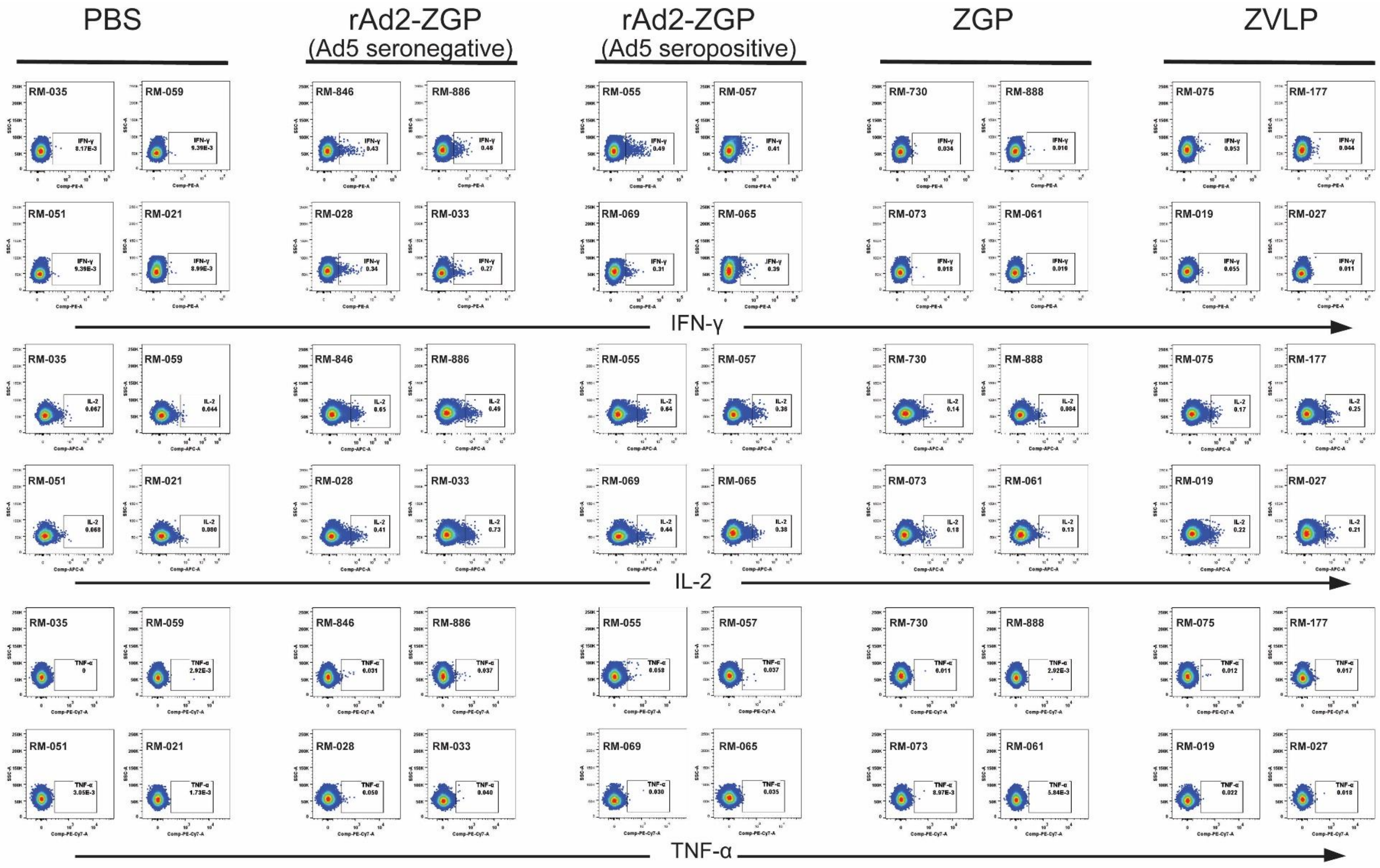

Supplementary Figure S5. Intracellular cytokine staining of CD8+ T cell from macaques immunized with EBOV vaccines. Chinese rhesus macaques were immunized intramuscularly with either 1×10^{11} vp rAd2-ZGP, or 400 µg ZGP, or 400 µg ZVLP, or PBS respectively (n=4). Two weeks after macaques received a booster immunization with the same vaccine, peripheral blood mononuclear cells were stimulated with a ZEBOV GP peptide pool and stained for specific markers (CD3, CD4, CD8, IFN-γ, IL-2, TNF-α). CD8+ T cells gated cells secreting GP-induced IFN-γ, IL-2, TNF-α for each macaque were determined.
