# Supplementary material for: An adenovirus serotype 2-vectored ebolavirus vaccine generates robust antibody and cell-mediated immune responses in mice and rhesus macaques
Source: Emerg Microbes Infect. 2018 Jun 6;7:101. doi: 10.1038/s41426-018-0102-5 (PMC5988821; doi:10.1038/s41426-018-0102-5)
Supplement: Supplementary file 7 — Supplementary Figure S6 [file 41426_2018_102_MOESM7_ESM.pdf]

Supplementary Figure S6

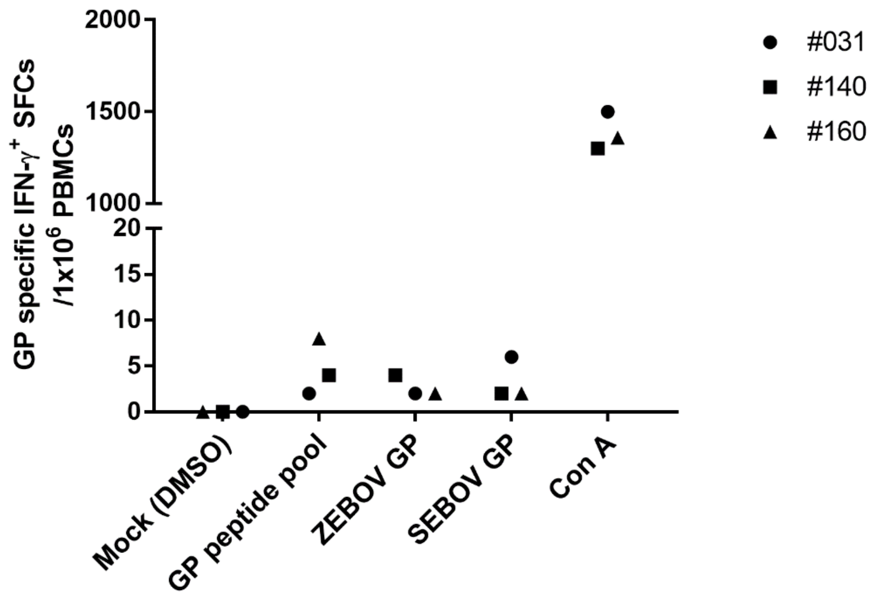

Supplementary Figure S6. GP specific IFN-γ^{+} ELISpot responses in PBMCs from macaques immunized with rAd2-zika.

Three Chinese rhesus macaques (#031, #140, #160) were immunized intramuscularly with 1×10^{11} vp rAd2-zika. Two weeks after immunization, IFN-γ^{+} ELISpot assays was conducted. PBMCs were isolated and stimulated with a ZEBOV GP peptide pool, ZEBOV GP or SEBOV GP. DMSO, the solvent of GP peptide pool, was used as a mock control and Con A was used as the positive control. Data were shown as the number of spot forming cells (SFCs) in one million PBMCs.
